# Restrictive annuloplasty or replacement on reverse remodeling for nonischemic dilated cardiomyopathy

**DOI:** 10.1186/s13019-024-02719-6

**Published:** 2024-04-12

**Authors:** Yusuke Misumi, Masashi Kawamura, Daisuke Yoshioka, Takuji Kawamura, Ai Kawamura, Yoshito Ito, Tsubasa Mikami, Masaki Taira, Kazuo Shimamura, Shigeru Miyagawa, Osaka Cardiovascular Surgery Research (OSCAR) study group

**Affiliations:** Department of Cardiovascular Surgery, Osaka University Graduate School of Medicine, 2-2-E1, Yamadaoka, Suita City, Osaka 565-0871 Japan

**Keywords:** Nonischemic dilated cardiomyopathy, Mitral annuloplasty, Mitral replacement, Left ventricular reverse remodeling

## Abstract

**Background:**

For patients with nonischemic dilated cardiomyopathy (NIDCM), the indications for and results of mitral surgery remain controversial. We reviewed a strategy of mitral repair and replacement for clinically relevant secondary mitral regurgitation (MR) in patients with NIDCM.

**Methods:**

We retrospectively reviewed 65 patients with advanced NIDCM (LVEF < 40%) who underwent mitral surgery. Of them, 47 (72%) underwent mitral annuloplasty and 18 (28%) replacement for secondary MR. The primary endpoint was postoperative reduction in indexed LV end-systolic volume (LVESVI).

**Results:**

At baseline, there was no intergroup difference in LVESVI (123 ± 47 vs. 147 ± 37 ml/m^2^, *P* = 0.055), LVEF (27 ± 8% vs. 25 ± 6%, *P* = 0.41), incidence of severe MR (57% (27/47) vs. 72% (13/18), *P* = 0.40), or EuroSCORE II score (6.2% vs. 7.6%, *P* = 0.90). At 6 months, the annuloplasty group reduced LVESVI to a greater degree than the replacement group (*P* < 0.001), yielding significantly smaller postoperative LVESVI (96 ± 59 vs. 154 ± 61 ml/m^2^, *P* < 0.001) and better LVEF (*P* < 0.001). The rates of moderate/severe recurrent MR were 17% (8/47) and 0%, respectively. Multivariable analysis demonstrated that mitral annuloplasty (OR 6.10, 95% CI 1.14–32.8, *P* = 0.035) was significantly associated with postoperative LV reverse remodeling. Cumulative survival was not different between the groups (*P* = 0.26).

**Conclusions:**

In patients with NIDCM, mitral annuloplasty reduced LV volume to a greater degree than did mitral replacement. These findings may assist with surgical options for secondary MR associated with NIDCM.

**Supplementary Information:**

The online version contains supplementary material available at 10.1186/s13019-024-02719-6.

## Background

In patients with nonischemic dilated cardiomyopathy, left ventricular (LV) reverse remodeling is strongly associated with better clinical outcomes [1–3]. Mitral regurgitation (MR), which is one of the major comorbidities associated with nonischemic LV dysfunction [4], adds preload to the left ventricle and potentially leads to excess volume loading and poor clinical outcomes [5–7].

For medically refractory functional MR, surgical correction of the valve is considered, but whether repair or replacement is the optimal approach remains controversial [8–12]. Provided that durable control of MR is accomplished, mitral valve (MV) repair seems to be associated with a greater degree of left ventricular (LV) reverse remodeling compared to MV replacement, with lower perioperative mortality [13–17]. MV replacement, on the other hand, provides durable correction of the valve with a lower risk of MR recurrence, an important predisposition to postoperative cardiac events, including heart failure and readmission. In ischemic etiology, evidence is accumulating, especially for those who are indicated for concomitant cardiac revascularization [18–26].

In nonischemic etiology, however, the clinical significance of surgical strategies for addressing MR remains unknown. Here, we reviewed the impact of mitral repair and replacement strategies on postoperative LV reverse remodeling and survival in patients with nonischemic cardiomyopathy.

## Methods

### Patients

This study retrospectively reviewed 65 patients with nonischemic cardiomyopathy (LV ejection fraction [LVEF] ≤ 40%) who underwent restrictive mitral annuloplasty (*n* = 47) or prosthetic valve replacement (*n* = 18) and who had a complete transthoracic echocardiogram at six months after surgery. All patients had functional moderate or severe MR caused by restricted leaflet closure. Mitral surgery was indicated for patients with symptomatic moderate or severe MR refractory to medical therapy. Patients with degenerative mitral disease, those with coronary artery disease, and those who underwent concomitant aortic valve surgery or left ventricular assist device implantation, redo or emergent surgery were excluded from this study. A flow diagram depicting the selection of the patients is illustrated in the Supplemental Figure.

### Surgical procedures

The surgical procedures were performed with conventional cardiopulmonary bypass with mild hypothermia. Myocardial protection was achieved by both antegrade and retrograde cold blood cardioplegia. The decision between MV repair and replacement was determined according to both the patients’ clinical profile, including cardiac function, and the surgeons’ experience. In 47 patients, the mitral valve was repaired with stringent restrictive mitral annuloplasty after careful assessments of the intercommissural distance and the height of the anterior leaflet. No other adjunct procedures were performed on the mitral valve itself. In 18 patients, the mitral valve was replaced with a biological prosthetic valve utilizing a posterior chordal-sparing technique. Tricuspid annuloplasty was concomitantly performed for patients with moderate or greater tricuspid regurgitation (TR) and/or a significantly dilated tricuspid annulus.

### Assessment of LV function and degree of MR

Two-dimensional and Doppler echocardiography procedures were performed prior to surgery (baseline) and six months after surgery to assess LV function and MR severity. LV volumes were measured with Teichholz method. The severity of MR was graded as 0 (absent), 1+ (trivial), 2+ (mild), 3+ (moderate), or 4+ (severe) based on color Doppler extent and spatial distribution of the regurgitant jet relative to the left atrial area. Recurrent MR was defined as MR ≥ 3 + grade at six months after surgery. None of the patients underwent implantation of cardiac support devices, such as pacemakers, which can independently lead to LV reverse remodeling, within six months after the operation.

### Follow-up and assessment of adverse events

After surgery, the patients were kept on standard heart failure medications, including angiotensin-converting enzyme inhibitors or angiotensin-II receptor blockers, beta-blockers, and diuretics. The primary endpoint of the study was postoperative LV reverse remodeling, which was assessed based on the LV end-systolic volume indexed to body surface area (LVESVI) using echocardiography at six months after surgery. The secondary endpoints were postoperative changes in LVEF, recurrent MR at six months after surgery, cumulative survival, and freedom from the composite of mortality or readmission for heart failure. Readmission for heart failure was defined as any hospitalization event due to heart failure after surgery. Follow-up was completed in all patients (100%) through a review of their clinical records for a median duration of 5.3 [interquartile range (IQR), 3.0–7.4] years.

### Statistical analysis

The quantitative data were tested for normality with the Shapiro‒Wilk test and presented as the mean ± standard deviation or median with IQR as appropriate. Normally distributed variables were compared with Student’s t test, whereas the Wilcoxon rank-sum test was used for nonnormal variables. Categorical variables are shown as frequencies with proportions and were compared using chi-square analysis or Fisher’s exact test, as appropriate. Postoperative changes in LVESVI and LVEF between the study groups were assessed with paired t tests. The associations of preoperative variables with postoperative LV reverse remodeling were examined with logistic regression analysis. The results are summarized as odds ratios (ORs), 95% confidence intervals (CIs), and *P* values. Calculation of cumulative survival and the composite of freedom from death and readmission for heart failure were performed using the Kaplan‒Meier method, and log-rank testing was performed to compare the groups. The associations of preoperative variables with cumulative survival were examined with Cox proportional hazard analysis. The results are summarized as hazard ratios (HR), 95% CIs, and *P* values. The multivariable model was analyzed with variables prespecified according to clinical relevance only, as shown in Table 1 and Supplemental Table. Statistical significance was determined as *P* < 0.05. JMP (Version 13; SAS Institute Inc., Cary, NC) software was used for statistical analysis.

**Table 1:** Logistic regression analysis for postoperative left ventricular reverse remodeling

Variables	Univariate			Multivariate		
	Odds ratio	95% CI	*p* value	Odds ratio	95% CI	*p* value
**Clinical data**						
Age	1.02	0.96–1.07	0.52	1.04	0.98–1.12	0.16
Male sex	0.69	0.22–2.14	0.52	1.17	0.29–4.76	0.83
Chronic kidney disease stage 4 or 5	0.90	0.29–2.76	0.85	0.79	0.19–3.34	0.75
Previous CRT	0.16	0.03–0.79	0.024	0.12	0.01–1.02	0.052
**Echocardiography**						
LV end-systolic volume index	0.98	0.97–0.99	0.001	0.99	0.98–1.01	0.27
LV ejection fraction	1.10	1.02–1.19	0.011	1.09	0.99–1.19	0.068
**Surgical data**						
Mitral annuloplasty	5.68	1.45–22.3	0.013	6.02	1.11–32.5	0.037
Tricuspid annuloplasty	2.29	0.78–6.69	0.13	1.10	0.27–4.51	0.89
Atrial maze	2.38	0.77–7.36	0.13	0.91	0.23–3.78	0.91

CI, confidence interval; CRT, cardiac resynchronization therapy; LV, left ventricle

## Results

### Patients

The baseline characteristics are summarized in Table 2. Patients who underwent MV annuloplasty were likely to present comparable baseline clinical conditions, which accounted for the same surgical risk indicated by the logistic EuroSCORE II (6.2 (IQR, 3.8–11.2) % vs. 7.6 (3.1–9.1) %, *P* = 0.90) compared with those undergoing MV replacement, except for the prevalence in chronic kidney disease stage 4 or 5 (34% (16/47) vs. 6% (1/18), *P* = 0.01). The annuloplasty group showed higher frequencies of concomitant atrial Maze procedures for atrial fibrillation than the replacement group (34% (16/47) vs. 6% (1/18), *P* = 0.01).

**Table 2:** Patient characteristics and surgical data

	Annuloplasty	Replacement	*p* value
Variables	(*n* = 47)	(*n* = 18)	
**Clinical data**			
Age, years	64 ± 9	63 ± 9	0.80
Male sex	34 (72%)	15 (83%)	0.34
Body surface area, m [^2^]	1.6 ± 0.2	1.6 ± 0.1	0.60
Medical history and presentation at baseline			
NYHA functional class III or IV	43 (91%)	17 (94%)	0.68
Logistic EuroSCORE II	6.2 [3.8–11.2]	7.6 [3.1–9.1]	0.90
Chronic kidney disease stage 4 or 5	16 (34%)	1 (6%)	0.010
Diabetes mellitus	13 (28%)	3 (17%)	0.34
Atrial fibrillation	24 (51%)	5 (28%)	0.086
Hypertension	12 (26%)	6 (33%)	0.53
Previous CRT	9 (19%)	5 (28%)	0.46
Medication at baseline			
Beta-blocker	35 (74%)	10 (83%)	0.51
ACE inhibitor or angiotensin-receptor blocker	27 (57%)	14 (82%)	0.057
Diuretic	40 (85%)	18 (100%)	0.032
Echocardiography at baseline			
LV end-diastolic volume index, ml/m^2^	162 ± 52	187 ± 42	0.082
LV end-systolic volume index, ml/m^2^	123 ± 47	147 ± 37	0.055
Ejection fraction, %	27 ± 8	25 ± 6	0.41
Left atrial dimension, mm	49 ± 8	57 ± 11	0.005
Mitral regurgitation grade			0.21
Moderate	21 (45%)	5 (28%)	
Severe	26 (55%)	13 (72%)	
Tricuspid regurgitation grade			0.55
Mild	20 (43%)	11 (65%)	
Moderate	14 (30%)	2 (12%)	
Severe	5 (11%)	2 (12%)	
Pulmonary artery systolic pressure, mmHg	45 ± 14	41 ± 9	0.32
**Surgical data**			
Papillary muscle approximation	22 (47%)		0.14
Chordal sparing		18 (100%)	0.92
Mitral ring/valve size			
24 mm	18 (38%)		
25 mm		1 (6%)	
26 mm	22 (47%)		
27 mm		10 (56%)	
28 mm	6 (13%)		
29 mm		6 (33%)	
30 mm	1 (2%)		
31 mm		1 (6%)	
Ring size, mm	26 ± 1	28 ± 1	
Concomitant procedures			
Tricuspid valve repair	33 (70%)	9 (50%)	0.13
Atrial maze	16 (34%)	1 (6%)	0.010
Surgical era			< 0.001
Before Jun. 2008	31 (66%)	2 (11%)	
After July. 2008	16 (34%)	16 (89%)	

Values are presented as mean ± standard deviation, median [interquartile range], or number (percentage) as shown. ACE, angiotensin-converting enzyme; CRT, cardiac resynchronization therapy; LV, left ventricle; NYHA, New York Heart Association

### Postoperative reduction in LV Dimension

**Fig. 1:**
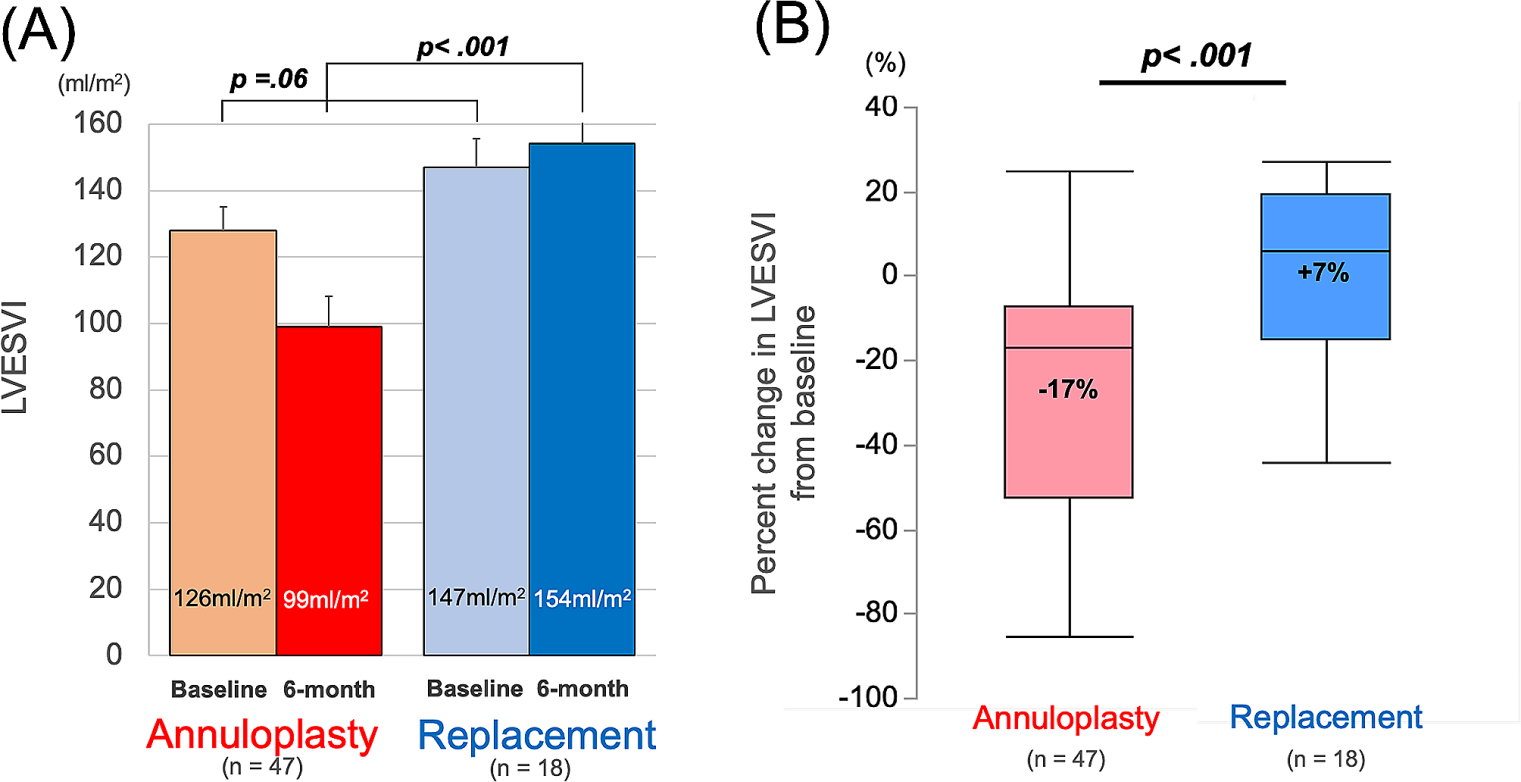
(**A**) Pre- and postoperative changes from the baseline of the left ventricular end-systolic dimension (LVESVI) and (**B**) box-plots showing percent changes in LVESVI. The box contains the 25th to 75th percentiles of the dataset, with the centerline denoting the median value. The whiskers mark the 5th and 95th percentiles

At preoperative baseline, the mean (± SD) LVESVI was not significantly different between the annuloplasty group and the replacement group (123 ± 47 vs. 147 ± 37 ml/m^2^, *P* = 0.055). At six months, the mean LVESVI was statistically smaller in the annuloplasty group (96 ± 59 vs. 154 ± 61 ml/m^2^, *P* < 0.001). The annuloplasty group had a greater reduction in LVESVI than the replacement group (*P* < 0.001). The median [IQR] absolute change and percent change from baseline were − 20 [-44 – (-8)] vs. +7 [-17 – (+ 35)] ml/m^2^ and − 17 [-53 - (-8)] vs. +8 [-14 - (+ 21)] % (Fig. 1).

### Associations between baseline characteristics and left ventricular reverse remodeling

When a reduction in LVESVI by ≥ 15% was defined as indicative of significant LV reverse remodeling [13, 27], 53% (*n* = 25 out of 47) and 17% (*n* = 3 out of 18) of patients who underwent MV annuloplasty and replacement achieved significant LV reverse remodeling, respectively (*P* = 0.006). Multivariable logistic regression analysis identified that MV annuloplasty (OR 6.02; 95% CI 1.11–32.5.; *P* = 0.037) was associated with greater LV remodeling (Table 1).

### Postoperative changes in ejection fraction and mitral regurgitation

**Fig. 2:**
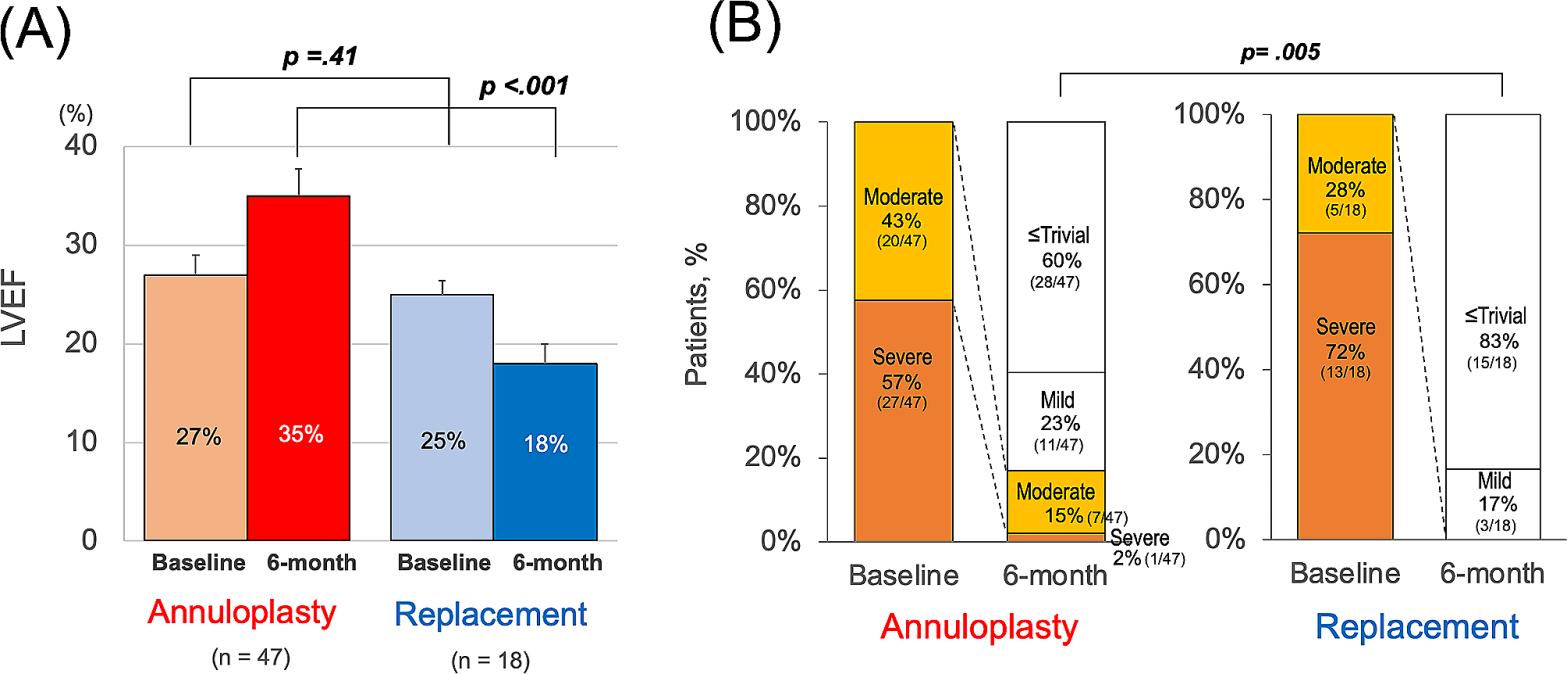
Pre- and postoperative changes in (**A**) left ventricular ejection fraction and (**B**) prevalence of moderate or greater mitral regurgitation

The LVEF was not significantly different between patients who underwent MV annuloplasty and replacement at preoperative baseline (27 ± 8% vs. 25 ± 6%, *P* = 0.41) but was statistically better in the annuloplasty group at six months postoperatively (36 ± 18% vs. 19 ± 8%, *P* < 0.001). The improvement in LVEF was significantly better in the annuloplasty group (*P* < 0.001). At baseline, MR grade was not significantly different between the annuloplasty group (43% had moderate and 57% had severe regurgitation) and the replacement group (28% had moderate and 72% had severe regurgitation) (*P* = 0.21). Six months postoperatively, the prevalence of significant MR (moderate or severe) was statistically higher in the annuloplasty group (annuloplasty vs. replacement, 17% (*n* = 8 out of 47) vs. 0% (*n* = 0 out of 18), *P* = 0.005) (Fig. 2).

### Early and long-term clinical outcomes

**Fig. 3:**
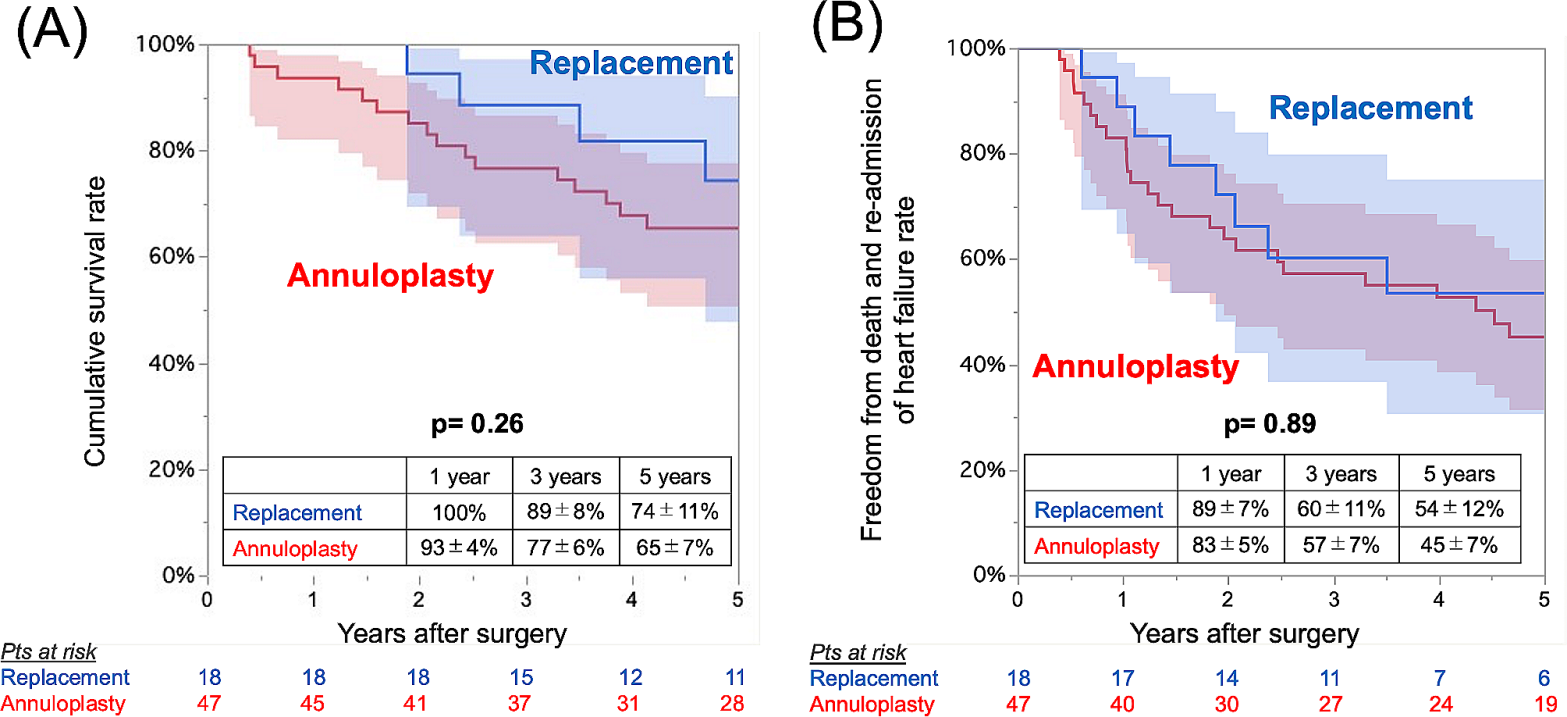
Kaplan‒Meier curves for (**A**) overall survival and (**B**) freedom from death and readmission for heart failure in all cohorts (*n* = 157) with 95% confidence intervals (shaded areas)

During follow-up, 27 (57%) and 7 (39%) patients died in the MV annuloplasty group and in the MV replacement group, respectively, and the cumulative survival rates at one, three, and five years were 93%, 77% and 65% and 100%, 89%, and 74%, respectively (log-rank *P* = 0.26). The rates of freedom from death or readmission for heart failure also did not differ between the groups (log-rank *P* = 0.89) (Fig. 3). Multivariable Cox proportional hazards analysis identified that concomitant mitral surgery (hazard ratio 2.10; 95% CI 0.37–12.0; *P* = 0.41) was not associated with cumulative survival (Supplemental Table).

One patient in the MV annuloplasty group presented with recurrent MR and underwent mitral replacement at 12 months after surgery. One patient in the MV replacement group presented with structural valve deterioration and underwent transcatheter valve-in-valve surgery seven years after surgery.

### Relationship between left ventricular reverse remodeling and survival in the annuloplasty group

**Fig. 4:**
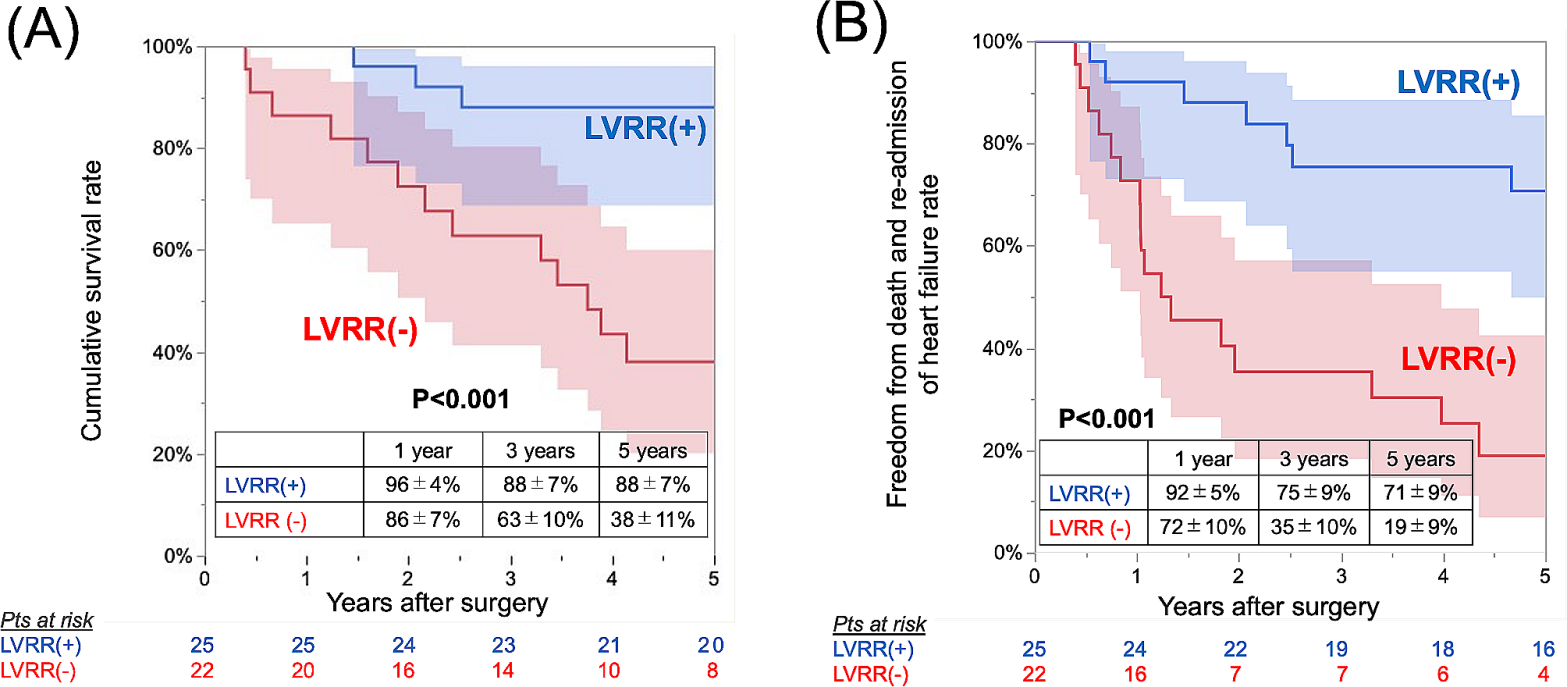
Kaplan‒Meier curves for (**A**) overall survival and (**B**) freedom from death or readmission for heart failure by postoperative left ventricular reverse remodeling in the mitral annuloplasty group with 95% confidence interval (shaded areas)

In the annuloplasty group, patients who achieved LV reverse remodeling had smaller LV dimensions, lower ejection fraction, and more advanced NYHA functional class and, consequently, a higher surgical risk, as indicated by logistic EuroSCORE II (Table 3). Univariable logistic regression analysis showed baseline NYHA functional class (*P* = 0.020), atrial fibrillation (*P* = 0.002), cardiac resynchronization therapy (*P* = 0.035), LV end-diastolic volume index (*P* = 0.029), and LVESVI (*P* = 0.011) were significantly associated with LV reverse remodeling. Multivariable analysis showed atrial fibrillation was significantly associated with LV reverse remodeling (OR 4.39, 95%CI 1.02–19.0, *P* = 0.048). The cumulative survival rate and freedom from composite events were significantly higher in patients with LV reverse remodeling. (Fig. 4).

**Table 3:** Patient characteristics and surgical data by postoperative left ventricular reverse remodeling in the mitral annuloplasty group

Annuloplasty			
LVRR		No LVRR	*p* value
Variables	(*n* = 25)	(*n* = 22)	
**Clinical data**			
Age, years	63 ± 9	64 ± 9	0.64
Male sex	18 (72%)	16 (73%)	0.96
Medical history and presentation at baseline			
NYHA functional class III or IV	21 (84%)	22 (100%)	0.020
Logistic EuroSCORE II	4.3 [2.6, 8.5]	8.0 [5.3, 13.5]	0.007
Chronic kidney disease stage 4 or 5	7 (28%)	9 (41%)	0.35
Atrial fibrillation	18 (72%)	6 (27%)	0.002
Diabetes mellitus	5 (20%)	8 (36%)	0.21
Hypertension	8 (32%)	4 (18%)	0.27
Previous CRT	2 (8%)	7 (32%)	0.035
Medication at baseline			
Beta-blocker	18 (72%)	17 (72%)	0.68
ACE inhibitor or angiotensin-receptor blocker	15 (60%)	12 (55%)	0.71
Diuretic	21 (84%)	19 (86%)	0.82
**Echocardiography**			
LV end-diastolic dimension, mm	67 ± 10	73 ± 10	0.039
LV end-systolic dimension, mm	58 ± 10	65 ± 11	0.018
LV end-diastolic volume index, ml/m^2^	147 ± 50	179 ± 50	0.033
LV end-systolic volume index, ml/m^2^	107 ± 43	141 ± 47	0.013
LV ejection fraction, %	29 ± 8	25 ± 8	0.065
Left atrial dimension, mm	50 ± 8	48 ± 9	0.33
Mitral regurgitation grade			0.49
Moderate	10 (40%)	11 (50%)	
Severe	15 (60%)	11 (50%)	
Tricuspid regurgitation grade			0.11
Mild	11 (44%)	9 (41%)	
Moderate	9 (36%)	5 (23%)	
Severe	3 (12%)	2 (9%)	
**Surgical data**			
Papillary muscle approximation	11 (44%)	11 (50%)	0.68
Concomitant procedures			
Tricuspid valve repair	20 (80%)	13 (59%)	0.12
Atrial maze	10 (40%)	6 (27%)	0.36

Values are presented as mean ± standard deviation, median [interquartile range], or number (percentage) as shown.ACE, angiotensin-converting enzyme; CRT, cardiac resynchronization therapy; LV, left ventricle; LVRR, left ventricular reverse remodeling; NYHA, New York Heart Association

### Relationship between left atrial dimension and clinical outcomes in the annuloplasty group

In the annuloplasty group, left atrial systolic dimension (LADs) significantly decreased from 49 ± 8 mm at baseline to 45 ± 9 mm at 6 month after surgery (*P* < 0.001). No statistical significant difference was observed in LADs between patients with LV reverse remodeling and those without both at baseline (50 ± 8 mm vs. 48 ± 9 mm, *P* = 0.33) and post-surgery 6-month (44 ± 8 mm vs. 46 ± 9 mm, *P* = 0.47). Preoperative LADs was not associated with LV reverse remodeling (OR 1.03, 95%CI 0.97–1.11, *P* = 0.33).

## Discussions

The major findings of this study can be summarized as follows. In a specific cohort of patients with nonischemic cardiomyopathy undergoing MV surgery, (i) patients who underwent MV annuloplasty had nearly identical degrees of LV remodeling at baseline, along with advanced grades of MR. (ii) They achieved a greater reduction in LVESVI, yielding significantly smaller LVESVI at six months postsurgery, compared with those undergoing MV replacement. (iii) The MV annuloplasty group demonstrated nearly identical long-term survival to the MV replacement group. Notably, we found that the MV annuloplasty procedure was independently associated with a greater reduction in postoperative LVESVI, a well-known predictor of mortality in patients with impaired LV function secondary to nonischemic etiology [1–3].

Although randomized clinical trials have addressed LV reverse remodeling following MV repair and replacement for functional mitral regurgitation, controversies still exist regarding the impact of the mitral procedure on postoperative LV reverse remodeling, presumably because the degree of baseline LV remodeling differed between the trials [18–26]. Acker and colleagues randomly assigned 251 patients with ischemic MR (mean LVEF 41%, LV end-systolic volume index (LVESVI) 64 ml/m^2^) to mitral annuloplasty or chordal-sparing replacement, resulting in no intergroup difference in postoperative reduction in LVESVI at one year postoperatively (percent change from baseline, -10% vs. -8%)^21^. However, another observational trial reported by De Bonis and colleagues confirmed the benefit of mitral repair [20]. They included 132 patients (mean LVEF 33%, LVESVI 79 ml/m^2^) who underwent restrictive annuloplasty or chordal-sparing replacement. At a median follow-up of 1.6 years postoperatively, the annuloplasty group demonstrated a greater reduction in LVESVI (percent change from baseline − 34% vs. -15%) than the replacement group. Our data appear consistent with the results from De Bonis and colleagues regarding the greater reduction in LV end-systolic volume, although the intergroup difference in the amount of LV end-systolic volume was modest. It is worth noting that the patients enrolled in our study presented more advanced stages of LV remodeling, as indicated by the larger LV volume and lower LVEF, than those enrolled in the previous study (e.g., LV end-systolic volume index; 126 ml/m^2^ vs. 82 ml/m^2^ for annuloplasty, 147 ml/m^2^ vs. 75 ml/m^2^ for replacement) [20]. Direct comparison of the findings among the studies may be difficult, given that the previous studies restricted their analysis to patients with severe functional MR, whereas our cohort was characterized by varying degrees of MR (i.e., among the annuloplasty group, 43% in moderate and 57% in severe grade) [21, 23]. Additionally, our cohort exclusively consisted of nonischemic etiology, whereas previous studies included patients with ischemic insult, with the rate of concomitant cardiac revascularization varying from 36 to 74% ^18,20,21,23^. These differences possibly contributed to better control of postoperative MR in the current study cohort (i.e., the rate of postoperative moderate or greater MR, 17% vs. 33%). However, the multivariable analysis in our study confirmed that the annuloplasty strategy was independently associated with a greater reduction in LVESVI, suggesting a potential benefit of MV annuloplasty for postoperative LV reverse remodeling in patients with advanced nonischemic cardiomyopathy.

Clinical studies have suggested that mitral replacement, compared with mitral repair, provides more durable correction of valvular lesions with a lower risk of recurrence, an important predisposition to postprocedural adverse cardiac events, including heart failure and readmission. However, controversies also exist regarding the impact of mitral repair and replacement strategies on postoperative survival and composite outcomes, presumably because the incremental degree of higher perioperative mortality associated with replacement differed between trials [18–26]. To primarily focus on the relationships between postoperative LV reverse remodeling and long-term clinical outcomes, our study included only patients who completed postoperative echocardiography at six months and thus eliminated the effect of early perioperative mortality on long-term results. Among our series of patients, those who underwent MV replacement achieved a significantly lower degree of postprocedural recurrent MR than those who underwent MV repair. However, no intergroup difference was shown in either cumulative survival or freedom from composite outcomes. Our results might be supported by findings from Ludwig and colleagues regarding an analysis of 262 patients with secondary mitral regurgitation (mean LVEF 39%, LV end-systolic volume (LVESV) 93 ml) who underwent transcatheter mitral edge-to-edge repair or replacement [28]. The transcatheter replacement group resulted in a more effective reduction in mitral regurgitation over the repair group (residual MR at discharge, 96% vs. 67%), but no between-group differences in mortality and composite endpoints were observed in a 30-day landmark analysis. The cumulative survival rates at one year were 80% after transcatheter replacement and 84% after transcatheter repair, which were comparable to the survival rates of the MV replacement group (100%) and the annuloplasty group (93%) in our study. Nevertheless, the impact of the mitral surgical strategy on long-term survival in patients with advanced cardiomyopathy remains to be determined.

There are some limitations to our study. First, this study was retrospective in nature and included a small number of subjects; thus, our results should be interpreted cautiously until verified in an independent, prospective study. Second, severity of MR was qualitatively assessed with color Doppler and not with quantitative methods such as regurgitant volume. Further investigation of a larger patient population with a longer follow-up is needed to definitively confirm our results.

## Conclusions

Patients who underwent MV annuloplasty had greater reductions in LV volume, yielding a significantly smaller LVESVI, than did those undergoing MV replacement. Further study is needed to identify the impact of LV reverse remodeling on survival.

### Electronic supplementary material

Below is the link to the electronic supplementary material.

Supplementary Material 1: Patient flow-chart

## Data Availability

The datasets used for this study are available from the corresponding author on reasonable request.

## Abbreviations

LV: Left ventricle
LVEF: Left ventricular ejection fraction.
LVESVI: Left ventricular end-systolic volume index.
MR: Mitral regurgitation.
MV: Mitral valve.
NIDCM: Nonischemic dilated cardiomyopathy.
RMA: Restrictive mitral annuloplasty.
