# Supplementary figures and images for: Restrictive annuloplasty or replacement on reverse remodeling for nonischemic dilated cardiomyopathy
Source: J Cardiothorac Surg. 2024 Apr 12;19:201. doi: 10.1186/s13019-024-02719-6 (PMC11010381; doi:10.1186/s13019-024-02719-6)

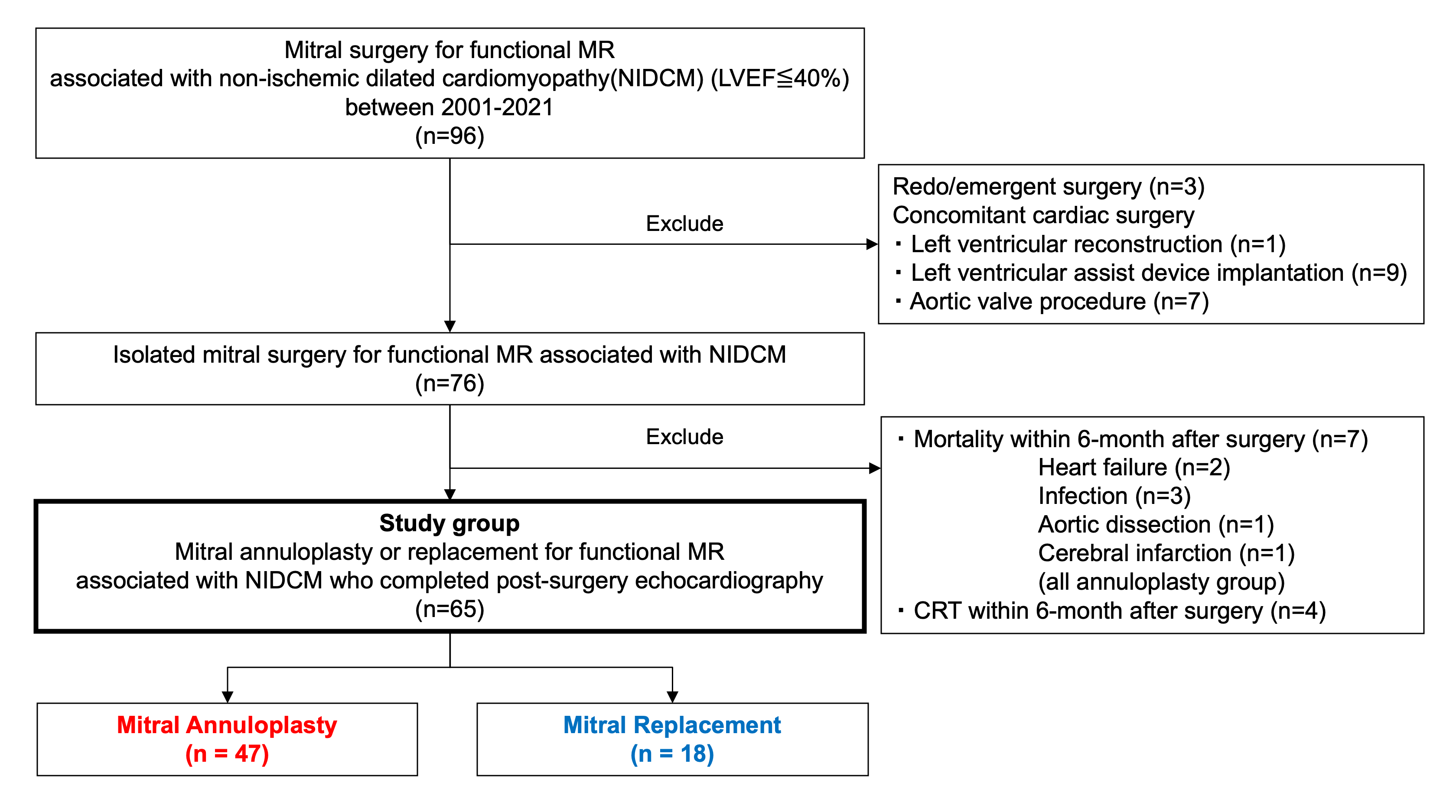

Supplement: Supplementary file 1 — Supplementary Material 1: Patient flow-chart [file 13019_2024_2719_MOESM1_ESM.png]
